# Radial Tiny Golden Angle UTE (tyGA‐UTE) for Cardiac MRI in the Small Animal Model

**DOI:** 10.1002/nbm.70418

**Published:** 2026-10-14

**Authors:** Hao Li, Patrick Metze, Pengxi Han, Benzheng Wei, Volker Rasche

**Affiliations:** ^1^ Department of Cardiology the First Affiliated Hospital of Shandong First Medical University & Shandong Provincial Qianfoshan Hospital Jinan China; ^2^ Center for Medical Artificial Intelligence Shandong University of Traditional Chinese Medicine Qingdao Shandong China; ^3^ Department of Internal Medicine II Ulm University Medical Center Ulm Germany; ^4^ Department of Radiology the First Affiliated Hospital of Shandong First Medical University & Shandong Provincial Qianfoshan Hospital Jinan China; ^5^ Ulm University Core Facility Small Animal Imaging Ulm Germany

**Keywords:** compressed sensing, imaging workflow optimization, mouse cardiac MRI, radial tiny golden angle (tyGA) MRI, self‐gating, sliding window, ultrashort echo time

## Abstract

Robust cardiorespiratory motion correction is critical for high‐quality ultra‐high‐field (UHF) preclinical cardiac magnetic resonance imaging (MRI). Whereas radial tiny golden angle (tyGA) sampling, data‐driven self‐gating (sg), compressed sensing (CS), and ultrashort echo time (UTE) are individually well‐established techniques, their integration into a unified acquisition and reconstruction pipeline, together with systematic validation at 11.7 T remains insufficiently explored. This study presents and validates a UHF‐optimized tyGA‐based UTE workflow that enables high‐temporal‐resolution sliding‐window dynamic imaging (SW‐tyGA‐UTE) and high‐spatial‐resolution cardiorespiratory self‐gated cine imaging (Gated‐tyGA‐UTE) from a single continuous acquired tyGA acquisition. All experiments were performed on an 11.7‐T small‐animal MRI system in 16 healthy wild‐type mice. Cardiac functional parameters, image quality metrics (signal‐to‐noise ratio [SNR] and contrast‐to‐noise ratio [CNR]), and inter‐/intra‐observer reproducibility were systematically compared between the proposed tyGA‐UTE pipeline and two navigator‐gated reference techniques: retrospectively gated FLASH and UTE methodologies. SW‐tyGA‐UTE and Gated‐tyGA‐UTE reconstructions yielded global left ventricular functional parameters comparable to those obtained with the reference methods (all *p* > 0.05). All functional metrics showed excellent intra‐observer (ICC = 0.94–0.99) and inter‐observer (ICC = 0.90–0.98) reproducibility, with ejection fraction showing the highest measurement consistency. Image quality was evaluated using an image‐based within‐ROI noise quantification approach that captures both thermal noise and reconstruction‐related artifacts. In this analysis, the Gated‐tyGA‐UTE protocol showed no statistically significant differences in left ventricular blood‐pool SNR, myocardial SNR, or CNR relative to the reference sequences. In contrast, the high‐temporal‐resolution sliding‐window reconstruction showed significantly reduced image quality across all three metrics, consistent with the expected spatiotemporal trade‐off of undersampled dynamic imaging. This study validates a robust, UHF‐optimized tyGA‐UTE imaging workflow that generates both high‐fidelity quantitative cine data and ultra‐fast dynamic cardiac images from a single acquisition. The unified pipeline eliminates the need for sequential repeated scans, minimizes anesthesia‐related physiological variability, and improves experimental efficiency, providing a practical tool for preclinical cardiac phenotyping, longitudinal studies, and drug screening.

## Graphical abstract

This study validates an 11.7‐T ultra‐high‐field tyGA‐UTE cardiac MRI workflow for murine models, featuring single‐acquisition dual reconstructions for real‐time visualization and quantitative functional assessment. It delivers performance equivalent to standard techniques, excellent reproducibility, and higher efficiency by eliminating repeated scans and reducing anesthesia variability.

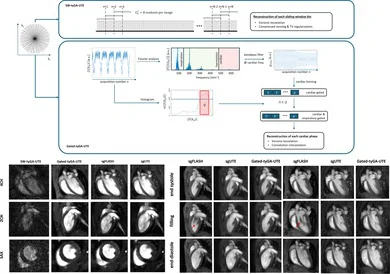

**Abbreviations**
2CH: two‐chamber long‐axis cardiac view
4CH: four‐chamber long‐axis cardiac view
CA: contrast agent
CI: confidence interval
CNR: contrast‐to‐noise ratio
CoV: coefficient of variation
CS: compressed sensing
ECG: electrocardiogram
EDV: end‐diastolic volume
EF: ejection fraction
ESV: end‐systolic volume
FLASH: fast low angle shot
ICC: intraclass correlation coefficient
LVMED: end‐diastolic left ventricular mass
LVMES: end‐systolic left ventricular mass
ML: machine learning
MRI: magnetic resonance imaging
PI: parallel imaging
PolyGate: spatially resolved multi‐animal self‐gating navigator framework
ROI: region of interest
SAX: short‐axis cardiac slice stack
SD: standard deviation
sg: self‐gating
SMS: simultaneous multi‐slice
SNR: signal‐to‐noise ratio
SV: stroke volume
swingGA: swing golden angle radial trajectory
TE: echo time
TR: repetition time
TV: total variation regularization
tyGA: radial tiny golden angle sampling trajectory
UHF: ultra‐high field
UTE: ultrashort echo time

## 1 Introduction

Cardiovascular disease remains the primary cause of global mortality, driving critical demand for precise in vivo phenotyping in murine models essential for mechanistic insights and therapeutic discovery [1, 2]. Magnetic resonance imaging (MRI) stands as the noninvasive reference standard for comprehensive cardiac phenotyping in these models, offering unparalleled soft‐tissue contrast for assessing ventricular structure, function, and tissue characterization [3]. The intrinsic challenge lies in mitigating artifacts induced by rapid physiological motions and blood flow, where cardiac rates exceeding 600 beats per minute (bpm) coexist with respiratory cycles surpassing 100 breaths/min [4, 5].

Conventional MRI strategies for mitigating cardiorespiratory motion primarily rely on prospective or retrospective gating with external physiological monitors (e.g., electrocardiogram [ECG] electrodes and pneumatic respiratory sensors) [6, 7]. Although widely used, these techniques have several important limitations in murine imaging, including ECG signal degradation at ultra‐high (UHF) magnetic field strengths, variable trigger delays that can disrupt the magnetization steady state, bulk motion of the animal, and reduced scan efficiency due to gating‐related acquisition overhead [8, 9].

Retrospective self‐gating strategies, for example, by use of additional navigator acquisitions [10, 11, 12], avoid hardware synchronization through repeated k‐space center sampling. Analysis of the k‐space center signal variation is usually used for data‐driven motion binning, which has become a quasi‐standard in UHF small animal cardiac imaging. Even though promising, this technique remains prone to artifacts in rapid murine cardiac imaging [7], for example, introduced by residual motion or incomplete filling of k‐space for each or individual motion bins. The combination with radial sampling greatly mitigates the motion‐induced artifacts and in combination with ultrashort echo time (UTE) strategies even reduce the flow‐related signal voids [13, 14], but the nonuniform k‐space coverage often still cause pronounced streak artifacts [15, 16]. With the introduction of the golden angle azimuthal increments (~111.25°), uniform k‐space filling even in case of severe undersampling was shown, yielding an excellent basis for flexible retrospective binning [17, 18] especially in combination with advanced reconstruction techniques such as compressed sensing (CS).

Joint cardiac and respiratory self‐gating derived from central k‐space data has also been established for radial cardiac imaging in clinical and preclinical settings [19, 20]. Despite these advances, conventional golden angle radial trajectories may suffer from eddy current artifacts especially when implemented at ultra‐high field (UHF). With tiny golden angle (tyGA) angular increments, a family of smaller irrational golden‐ratio‐based angular increments were introduced to reduce eddy‐current‐related artifacts while preserving flexible retrospective reconstruction [21]. Combined with advanced reconstruction techniques [22, 23, 24, 25], gradient echo tyGA acquisitions have demonstrated promising results for ungated high‐temporal sliding‐window imaging in mice. Temporal resolutions below 20 ms were reported, which allow assessment of beat‐to‐beat motion differences as well as first‐pass myocardial perfusion [26]. Because temporal resolution is prioritized, spatial resolution and image quality are compromised [26], and usually, an additional high‐spatial‐resolution cine scan for accurate quantification of ventricular structure and function is acquired. Therefore, the related prolonged anesthesia time increases the likelihood of fluctuations in heart and respiratory rates as well as the overall burden on the animal.

Considering the complementary properties of the aforementioned techniques, the aim of this work was to investigate whether a single continuous tyGA‐UTE acquisition can provide both high‐temporal‐resolution sliding‐window images and self‐gated high‐spatial‐resolution cine images while simultaneously reducing flow‐related signal loss and associated artifacts.

We therefore developed and systematically validated a tyGA‐UTE workflow providing two complementary image series from a single continuous acquisition. The performance of the proposed approach was benchmarked against two established navigator‐gated reference standards (gated FLASH and gated UTE) in a 16‐mouse in vivo cohort, with comprehensive evaluation of functional accuracy, image quality, and measurement reproducibility.

## 2 Methods

### 2.1 Animals

#### 2.1.1 Animals and Ethics Considerations

A total of 16 male wild‐type mice (C57BL/6N, 26–30 g) were included in this study. Before the start of the study, the animals were housed in a temperature‐controlled environment for at least 1 week. Facility rooms were maintained at constant temperature (23°C), humidity (50% relative humidity), and 12‐h light/dark cycle. Access to food and tap water was ad libitum.

Animal experiments were approved by the regional board of Tübingen and conducted according to German law for the welfare of animals and regulations for care and use of laboratory animals. All institutional and national guidelines for the care and use of laboratory animals were followed and approved by the appropriate institutional committees.

#### 2.1.2 Anesthesia of the Animals

All mice were anesthetized with inhaled isoflurane using 5% in medical air at 0.1 L/min for induction and 1.0%–1.5% for maintenance during imaging. Animals were positioned prone on a circulating warm‐water pad, and body temperature was continuously monitored and maintained within ±0.5°C. Respiratory motion was recorded using a pneumatic balloon sensor placed against the abdominal wall, whereas the isoflurane concentration was adjusted to maintain a respiratory rate of 100–120 breaths/min.

### 2.2 CMR Image Acquisition

Sixteen mice underwent a three‐protocol examination comprising navigator‐based self‐gated FLASH (sgFLASH), self‐gated UTE (sgUTE), and tyGA‐UTE acquisitions. Imaging was performed on an 11.7‐T preclinical MRI system (BioSpec 117/16, Bruker BioSpin, Ettlingen, Germany) equipped with gradients providing a maximum amplitude of 760 mT/m and a peak slew rate of 6840 T/m/s. Excitation was performed with a 72‐mm quadrature volume coil and signal was received with a dedicated four‐channel thoracic phased‐array coil [27](RAPID Biomedical, Rimpar, Germany).

Planning of the scan geometry followed a previously described standardized workflow [28] to ensure reproducible imaging geometry across examinations. Briefly, axial, coronal, and sagittal survey images were acquired first, followed by semi‐two‐chamber and semi four‐chamber cine views used for planning of eight contiguous short‐axis slices fully covering the left ventricle (LV) in end‐diastole, which was then acquired with the three investigated sequences (sgFLASH, sgUTE, and tyGA‐UTE) in random order. Please refer to Table 1 for detailed acquisition parameters and to Data S1 and S2 for a detailed description of the planning procedure.

**TABLE 1:** Acquisition parameters for the investigated retrospectively gated FLASH (sgFLASH), retrospectively gated UTE (sgUTE), and tyGA techniques (tyGA‐UTE).

	tyGA‐UTE	sgFLASH	sgUTE
TE/TR (ms)	0.25/2.36	1.04/5.62	0.25/3.20
Flip angle (deg)	15	15	15
Matrix size	256 × 256	256 × 256	256 × 256
Field of view (mm^2^)	30 × 30	30 × 30	30 × 30
Spatial resolution (mm^2^)	0.117 × 0.117	0.117 x 0.117	0.117 × 0.117
Slice thickness (mm)	1	1	1
Angular increment (deg)	≈23.63 ($\psi_{7}$)	NA	≈0.45
# oversampling	40	200	80
Half‐k‐space	NA	Yes, 20% central oversampling	NA
Bandwidth (kHz)	250	125	250
T_ACQ_/slice (s)	77 s	157 s	206 s

In particular, the following sequences were acquired in all animals (for sequence details, please refer to Table 1).

- sgFLASH: Cartesian k‐space encoding (IntraGate, ParaVision 6.0.1, Bruker Biospin). The sgFLASH is the current preclinical reference for high‐resolution protocol: echo/repetition time (TE/TR) = 1.04/5.62 ms, 20 frames per cardiac cycle, oversampling = 200, half‐k‐space acquisition with 20% central oversampling, acquisition time [T_ACQ_] = 2 min 37 s per slice.
- sgUTE: Radial center‐out k‐space encoding with linear angular increment $∆\theta \approx 0.45^{{}^{\circ}}$, TE/TR = 0.25/3.20, 20 frames per cardiac cycle, projections = 804, oversampling = 80, T_ACQ_ = 3 min 26 s per slice.
- tyGA‐UTE: Radial center‐out k‐space encoding with tiny golden angle angular increment [29] $∆\theta =\psi_{7}\approx 23.63^{{}^{\circ}}$, TE/TR = 0.25/2.36 ms, projections = 32,358, T_ACQ_ = 1 min 17 s per slice.

For all sequences, the minimal possible repetition time was used to minimize the overall acquisition time (T_ACQ_) per slice. In comparison with tyGA‐UTE (TR = 2.36 ms), the longer repetition times reported for sgFLASH (5.62 ms) and sgUTE (3.20 ms) are caused by additional navigator signal acquisition prior to each imaging readout.

For all radial acquisitions, the actual center‐out k‐space trajectory was mapped individually in each animal using a phase‐based trajectory mapping approach [30] adopted by the vendor of the MR system. The mapped trajectories were exported and fully considered during offline image reconstruction. Furthermore, in the UTE scans excitation was performed using an asymmetric Sinc pulse comprising the central lobe plus one leading lobe.

For sgFLASH and sgUTE, the required slab for deriving the navigator data was positioned to encompass the complete LV with a flip angle of 2°.

### 2.3 Data Reconstruction

sgFLASH and sgUTE reference data were reconstructed by the navigator‐based retrospective self‐gating software as provided by the vendor (IntraGate, ParaVision 6.0.1, Bruker Biospin, Ettlingen, Germany). Based on the obtained navigator signals, images were reconstructed in a single respiratory stage and 20 cardiac phases equally spread over the cardiac cycle.

tyGA‐UTE data were processed using an in‐house reconstruction framework providing flexible sliding‐window and gated reconstruction [31], solely based on the imaging data (Figure 1), implemented in Matlab (Matlab, The MathWorks, Natick, Massachusetts, USA).

**FIGURE 1:**
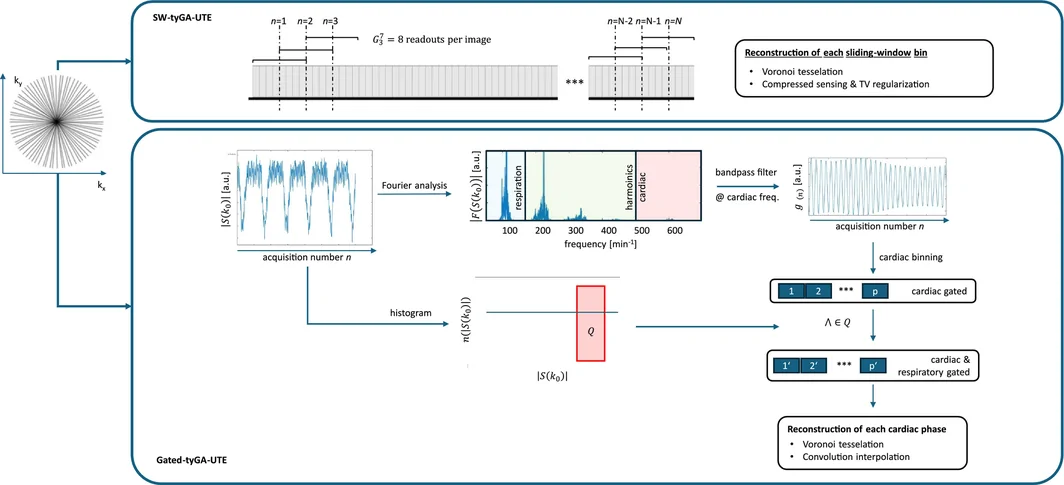
Reconstruction workflow: From the continuously acquired tyGA‐UTE datasets, two complementary reconstruction pipelines were used: (1) high‐temporal‐resolution sliding‐window reconstruction (SW‐tyGA‐UTE) and (2) high‐fidelity k‐space‐based self‐gated cine reconstruction (Gated‐tyGA‐UTE).

In the sliding‐window reconstruction framework (SW‐tyGA‐UTE) [32], the temporal resolution $∆T_{\mathrm{bin}}$ (number of projections per bin times TR) and temporal offset $∆T_{\text{shift}}$ (number of projections between consecutive reconstruction times TR) can be freely adjusted. For mitigating aliasing artifacts, a CS scheme with spatiotemporal total variation (TV) regularization, formulated as [30]

argminF·S·x−b22+λt∇tx1+λs∇sx1,

is used for reconstruction, with F denoting the nonuniform Fourier transform operator mapping between Cartesian x−t to radial k−t space and S containing all coil sensitivities. The $l_{2}$‐norm enforces data consistency between k−t space samples *b* and the reconstructed image sequence *x* in x−t space. $\nabla_{s}$ and $\nabla_{t}$ denote the gradient operator along the spatial and temporal dimension. In all reconstructions, 50 iterations were performed with fixed regularization strengths: $\lambda_{s}=0.05$ for spatial and $\lambda_{t}=0.005$ for temporal constraints. For estimation of S, low‐resolution images $I_{\mathrm{LR}}^{i}$ were reconstructed from the central 25% of k‐space averaged over all acquired projections individually for each coil *i*. Coil‐specific sensitivity maps $S_{i}$ were then calculated as Si=∑jILRj2ILRi.

All SW‐tyGA‐UTE data were reconstructed from $G_{3}^{7}=8$ readouts, yielding a nominal temporal resolution of $∆T_{\mathrm{bin}}$ = 18.88 ms, where $G_{3}^{7}$ denotes the third element of the generalized Fibonacci sequence associated with the seventh tiny golden angle $\psi_{7}$, corresponding to an optimal k‐space coverage with eight consecutive projections. Images were reconstructed with $∆T_{\text{shift}}=9.44\mathrm{ms}$ (4 readouts).

For Gated‐tyGA‐UTE, the self‐gating information was extracted solely from the magnitude of the k‐space center signal $S\left(k_{0}\right)$. For identification of the actual respiratory and cardiac frequencies of the animal, a Fourier analysis of the signal variations of the $S\left(k_{0}\right)$ signal was performed. A cardiac gating signal was generated from the $S\left(k_{0}\right)$ data by applying a Butterworth bandpass filter with stop band attenuation of 30 dB at the identified cardiac frequencies. Considering the respiratory pattern of the mouse (short inspiration with rather long stable expiration phase), respiratory quiescence phases were identified by histogram analyses of the unfiltered $S\left(k_{0}\right)$ data, with assigning the upper 30% of the histogram to the quiescent phase of the respiration. Final reconstruction of the gated data was performed separately for each cardiac bin applying a convolution interpolation (Gridding) with full consideration of the resulting individual nonuniform coverage of k‐space by identification of the local sampling density by Voronoi tessellation [33].

### 2.4 Data Analysis

#### 2.4.1 Assessment of LV Function

Left ventricular functional parameters were analyzed using Segment 4.1.0.1 R14284b (http://segment.heiberg.se). Both endocardial and epicardial contours were initially automatically identified by the built‐in algorithm, followed by manual review and correction. End‐diastolic volume (EDV), end‐systolic volume (ESV), stroke volume (SV), ejection fraction (EF), left ventricle mass at end‐diastole (LVMED), and left ventricle mass at end‐systole (LVMES) were quantified. For assessment of the intra‐observer variability, the primary observer reanalyzed all images after a 2‐week interval in random order. For assessment of the inter‐observer variability, the data were independently analyzed by a second reviewer.

#### 2.4.2 Data Quality Analysis

The signal‐to‐noise ratio (SNR) in the myocardium and the LV blood and the contrast‐to‐noise ratio (CNR) between myocardium and LV blood were quantified in all animals for all investigated protocols (SW‐tyGA‐UTE, Gated‐tyGA‐UTE, sgFLASH, and sgUTE) in a mid‐ventricular slice during end‐diastole. Given that CS reconstruction employs TV regularization, which nonlinearly suppresses background noise and renders conventional air‐background noise measurements unreliable for CS‐reconstructed images [34, 35], we employed an image‐based within‐ROI method to quantify SNR and CNR. In this approach, noise is estimated from the signal variation within the tissue itself, thereby capturing both thermal noise and reconstruction‐related artifacts (e.g., streaking from undersampling). Specifically, circular ROIs were placed in the left ventricular blood pool (avoiding papillary muscles and endocardial borders) and in the septal myocardium (avoiding partial volume effects at the blood–myocardium and myocardium–epicardium interfaces). The image‐based SNR was calculated as ${\mathrm{SNR}}_{\text{blood}}=\frac{\mu_{\text{blood}}}{\sigma_{\text{blood}}}$ and ${\mathrm{SNR}}_{\mathrm{myo}}=\frac{\mu_{\mathrm{myo}}}{\sigma_{\mathrm{myo}}}$, where μ and σ denote the mean signal intensity and standard deviation within the respective ROI. The CNR was calculated as $\mathrm{CNR}=\left|\mu_{\text{blood}}-\mu_{\mathrm{myo}}\right|/\sqrt{\sigma_{\text{blood}}^{2}+\sigma_{\mathrm{myo}}^{2}}$.

#### 2.4.3 Statistical Analysis

All image analyses were done blinded and assessed quantitative parameters were presented as mean ± standard deviation (SD). Statistical significance of the differences between the investigated MR protocols was assessed by applying a Friedman test. Differences were considered statistically significant with *p*‐values ≤ 0.05. The inter‐ and intra‐observer reproducibility were assessed using the intraclass correlation coefficient (ICC), coefficient of variations (CoVs), and Bland–Altman analysis. Agreement was considered excellent for ICC > 0.74, good for ICC 0.6–0.74, fair for ICC 0.40–0.59, and poor for ICC < 0.40.

## 3 Results

Navigator‐based retrospectively gated FLASH, retrospectively gated UTE, and tyGA‐UTE could be completed in all 16 mice. Representative image quality across protocols is demonstrated in Figure 2, showing robust anatomical delineation of cardiac chambers and surrounding thoracic structures with all investigated approaches. Respective sliding‐window (SW‐tyGA‐UTE) and cine (sgUTE, sgFLASH, and Gated‐tyGA‐UTE) dynamic data are shown in the Supporting Information. The vascular structures as well as the LV can be well appreciated, and quantitative assessments of the functional parameters were possible even for the high‐temporal resolution‐sliding‐window reconstruction. The gated tyGA‐UTE sequence demonstrated reduction in flow artifacts compared to conventional gated FLASH acquisitions, particularly during ventricular filling phases (Figure 3). This mitigation of signal voids benefits cardiac chamber boundary delineation. Quantitative analysis revealed no statistically significant differences (*p* > 0.05) between conventional retrospectively gated (sgFLASH/sgUTE) and tyGA‐UTE‐derived (SW‐tyGA‐UTE/Gated‐tyGA‐UTE) protocols for any evaluated metric, as detailed in Figure 4 and Table 2.

**FIGURE 2:**
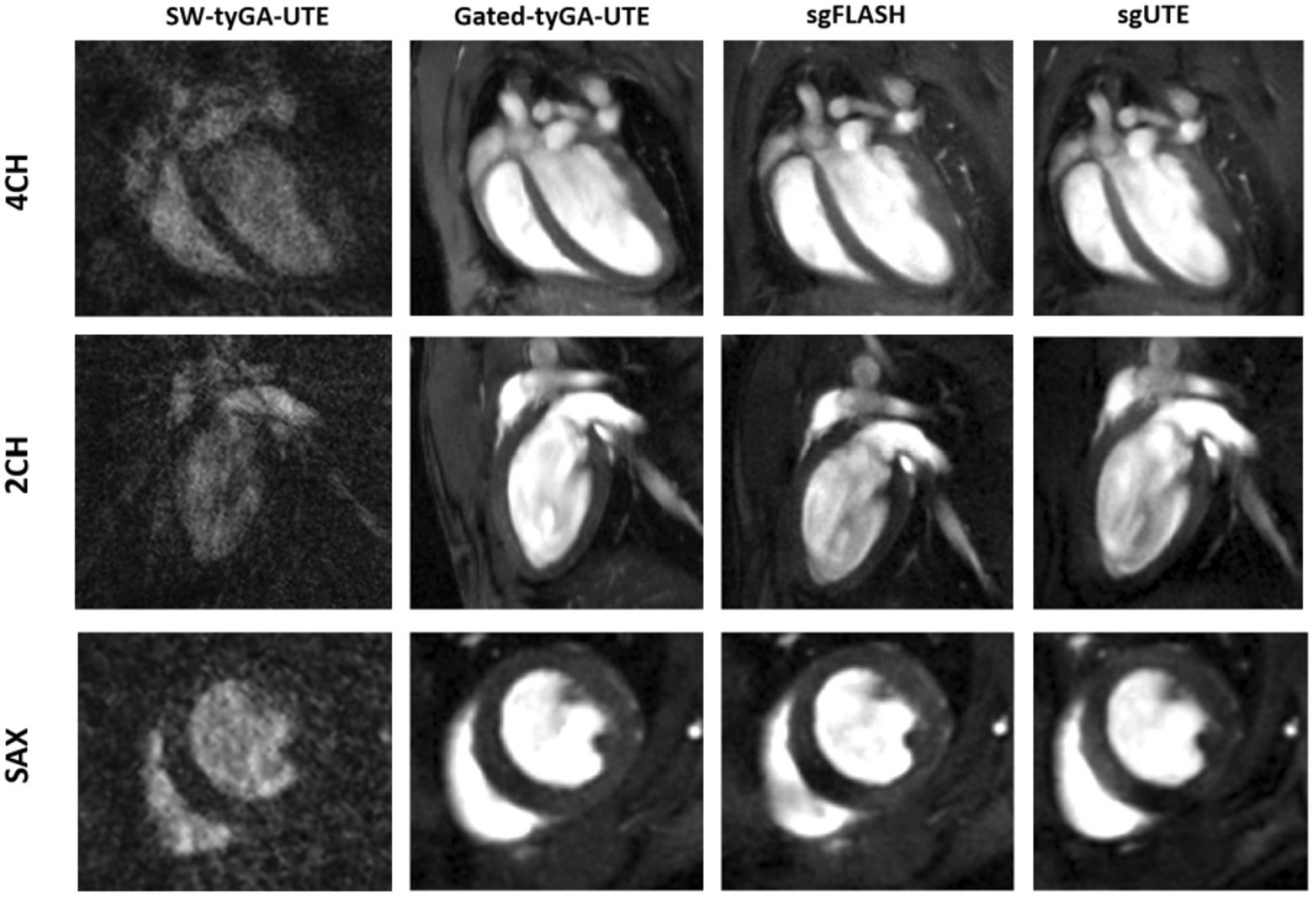
Comparison of the retrospectively gated FLASH (sgFLASH), retrospectively gated UTE (sgUTE), and tyGA‐UTE with high‐temporal‐resolution sliding‐window retrospective dynamic (SW‐tyGA‐UTE) and self‐gated (Gated‐tyGA‐UTE) reconstruction methodologies.

**FIGURE 3:**
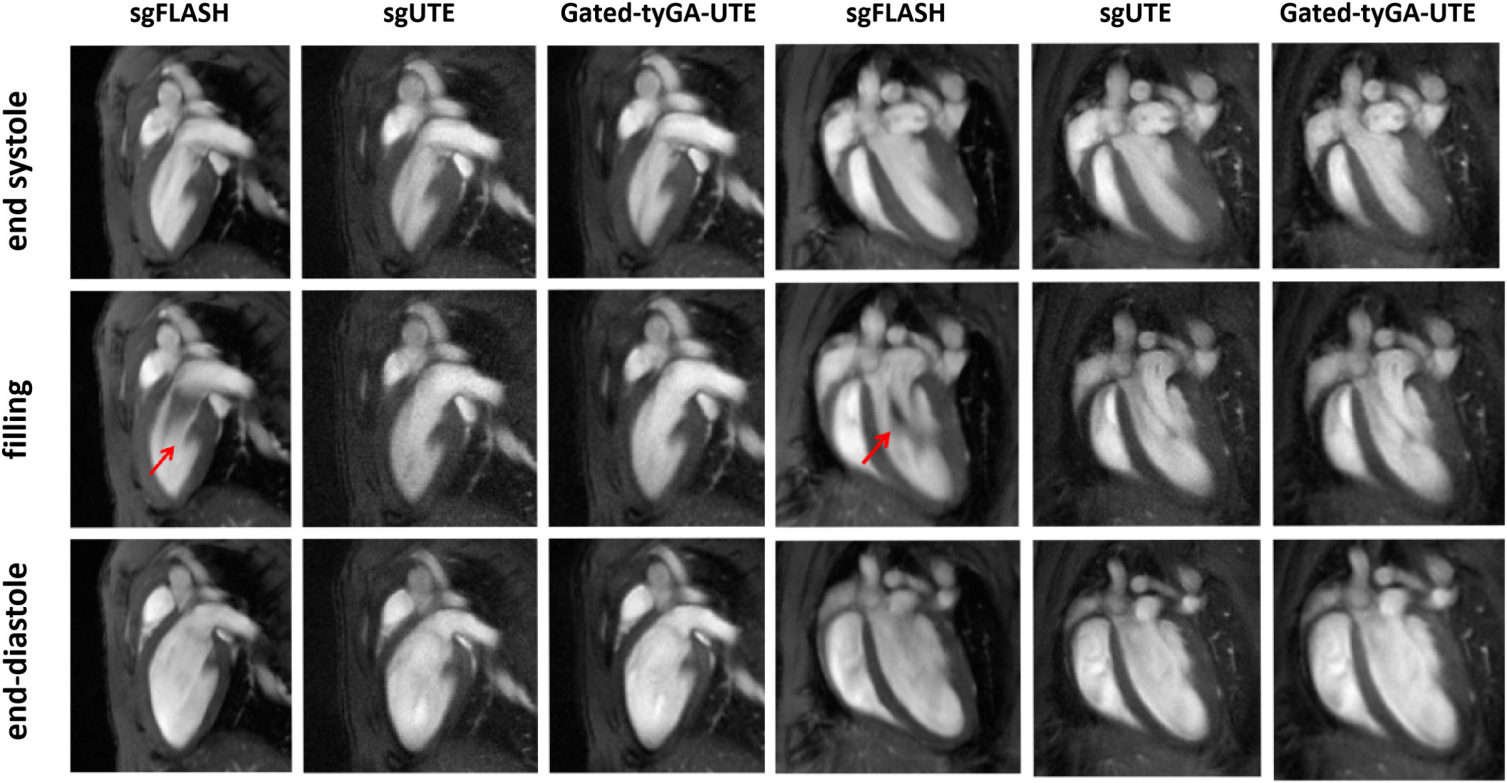
Comparison of intraventricular flow artifacts (red arrow) across gated MRI sequences at different cardiac phases.

**FIGURE 4:**
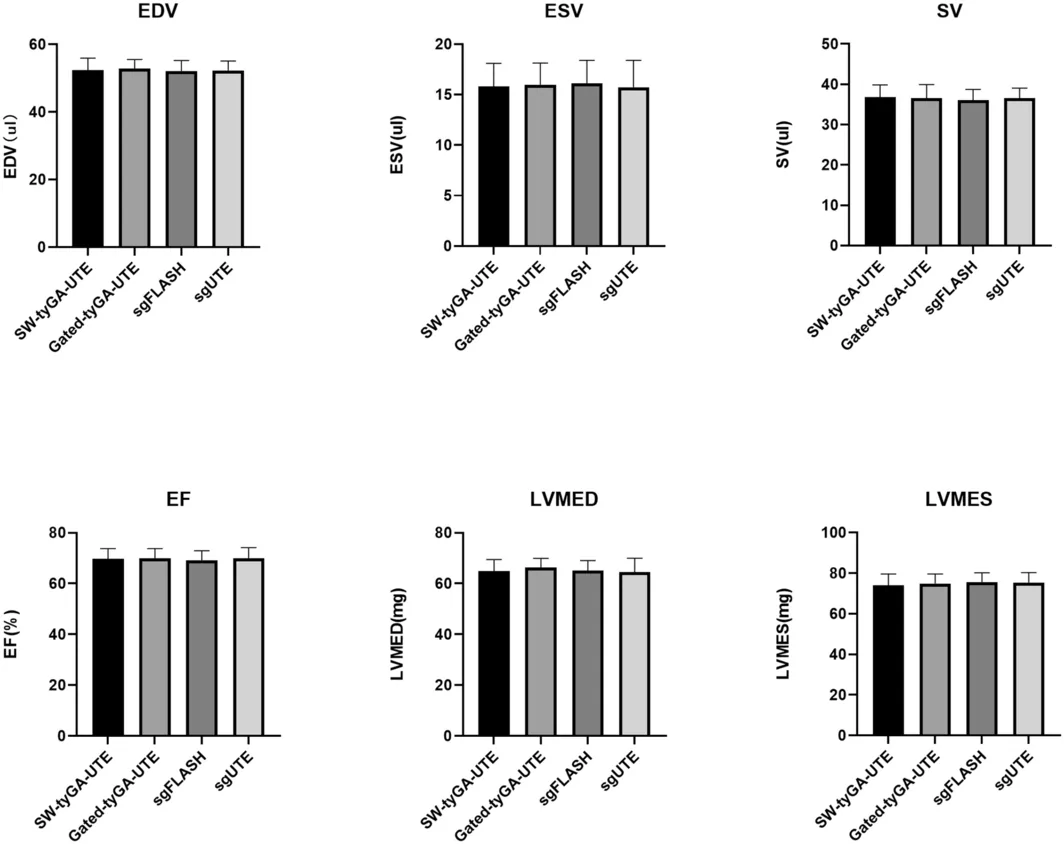
Comparison of cardiac function parameters (EDV/ESV, end‐diastolic/systolic volume; SV, stroke volume; EF, ejection fraction) and left ventricular mass (LVMED/ES, end‐diastolic/systolic left ventricular mass) for the investigated retrospectively gated FLASH (sgFLASH), retrospectively gated UTE (sgUTE), and tyGA‐UTE with high‐temporal‐resolution sliding‐window retrospective dynamic (SW‐tyGA‐UTE) and self‐gated (Gated‐tyGA‐UTE) reconstruction methodologies.

**TABLE 2:** Comparison of LV functional parameters for the investigated retrospectively gated FLASH (sgFLASH), retrospectively gated UTE (sgUTE), and tyGA‐UTE with high‐temporal‐resolution sliding‐window retrospective dynamic (SW‐tyGA‐UTE) and self‐gated (Gated‐tyGA‐UTE) reconstruction methodologies.

Parameters	SW‐tyGA‐UTE	Gated‐tyGA‐UTE	sgFLASH	sgUTE	*p*
EDV (μL)	52.40 ± 3.55	52.83 ± 2.71	52.11 ± 3.11	52.28 ± 2.79	0.68
ESV (μL)	15.81 ± 2.27	15.97 ± 2.16	16.09 ± 2.31	15.72 ± 2.66	0.70
SV (μL)	36.86 ± 3.00	36.59 ± 3.34	36.03 ± 2.70	36.55 ± 2.52	0.43
EF (%)	69.74 ± 4.03	69.81 ± 3.99	69.16 ± 3.73	69.98 ± 4.20	0.48
LVMED (mg)	64.81 ± 4.61	66.21 ± 3.66	65.00 ± 4.03	64.48 ± 5.49	0.69
LVMES (mg)	74.06 ± 5.58	74.85 ± 4.78	75.50 ± 4.73	75.38 ± 4.88	0.14

*Note:* Results are shown as mean ± SD.

Abbreviations: EDV/ESV = end‐diastolic/systolic volume, EF = ejection fraction, LVMED/ES = end‐diastolic/systolic left ventricular mass, SV = stroke volume.

### 3.1 Intra‐Observer and Inter‐Observer Variability

Intra‐observer reproducibility (Table 3 and Data S3) shows excellent measurement consistency for all functional parameters. The relative mean percentage differences between two 2‐week‐separated repeated measurements ranged from −0.18% to 0.69%. ICC values across all parameters ranged from 0.94 (95% CI: 0.87–0.97) to 0.99 (95% CI: 0.98–1.00), and CoVs were all below 2.2%. EF achieved the highest intra‐observer consistency with an ICC of 0.97 and the minimal CoV of 0.90%, whereas SV showed the widest 95% agreement interval (−6.50%–4.96%) and the largest intra‐observer CoV of 2.11%.

**TABLE 3:** Intra‐observer reproducibility of left ventricular functional metrics derived from tyGA‐UTE data reconstructed via high‐temporal‐resolution sliding‐window retrospective dynamic and self‐gated cine pipelines.

Parameter	S1	S2	Mean difference (%)	Limits of agreement (%)	ICC (95% CI)	Coefficient of variations (%)
EDV	52.62 ± 3.11	52.47 ± 3.17	0.47	−3.48–3.78	0.97 (0.95–0.99)	1.33
ESV	15.89 ± 2.18	15.81 ± 2.13	0.49	−2.08–3.88	0.99 (0.98–1.00)	1.25
SV	36.72 ± 3.12	36.66 ± 3.13	0.69	−6.50–4.96	0.94 (0.87–0.97)	2.11
EF	69.77 ± 3.94	69.85 ± 3.84	0.12	−3.03–2.00	0.97 (0.95–0.99)	0.90
LVMED	65.51 ± 4.16	65.59 ± 4.51	−0.18	−3.07–4.81	0.96 (0.92–0.98)	1.35
LVMES	74.46 ± 5.13	74.56 ± 5.28	0.02	−4.65–3.60	0.96 (0.92–0.98)	1.40

*Note:* Results are shown as mean ± SD.

Abbreviations: CI = confidence interval, EDV/ESV = end‐diastolic/systolic volume, EF = ejection fraction, ICC = intraclass correlation coefficient, LVMED/ES = end‐diastolic/systolic left ventricular mass, SV = stroke volume.

For inter‐observer reproducibility (Table 4 and Data S4), relative mean percentage differences ranged between −0.85% and 0.37%. All ICC values exceeded 0.90, and all CoV values were below 3.0%. Consistent with intra‐observer outcomes, EF demonstrated the highest inter‐observer reproducibility, with a tiny relative mean difference of 0.37%, tight 95% limits of agreement (−3.61%–3.93%), and the minimum CoV of 1.23%. ESV maintained strong inter‐operator consistency (ICC = 0.98, CoV = 1.87%), whereas SV displayed the broadest 95% limits of agreement (−8.23%–8.19%) and the largest inter‐observer CoV (2.94%).

**TABLE 4:** Inter‐observer reproducibility of left ventricular functional metrics derived from tyGA‐UTE data reconstructed via high‐temporal‐resolution sliding‐window retrospective dynamic and self‐gated cine pipelines.

Parameter	S1	S2	Mean difference (%)	Limits of agreement (%)	ICC (95% CI)	Coefficient of variations (%)
EDV	52.62 ± 3.11	52.51 ± 3.59	−0.62	−5.17–4.89	0.91 (0.82–0.96)	1.91
ESV	15.89 ± 2.18	15.96 ± 2.29	−0.43	−5.74–4.23	0.98 (0.96–0.99)	1.87
SV	36.72 ± 3.12	36.54 ± 3.51	−0.78	−8.23–8.19	0.90 (0.80–0.95)	2.94
EF	69.77 ± 3.94	69.56 ± 4.08	0.37	−3.61–3.93	0.97 (0.95–0.99)	1.23
LVMED	65.51 ± 4.16	65.74 ± 4.68	−0.85	−5.83–5.52	0.91 (0.83–0.96)	2.03
LVMES	74.46 ± 5.13	74.46 ± 5.43	0.10	−5.83–5.73	0.91 (0.83–0.96)	2.09

*Note:* Results are shown as mean ± SD.

Abbreviations: CI = confidence interval, EDV/ESV = end‐diastolic/systolic volume, EF = ejection fraction, ICC = intraclass correlation coefficient, LVMED/ES = end‐diastolic/systolic left ventricular mass, SV = stroke volume.

### 3.2 Data Quality Analysis

Gated‐tyGA‐UTE showed statistically equivalent (Figure 5) blood pool SNR, myocardial SNR and CNR relative to both sgFLASH and sgUTE reference sequences (blood pool SNR: 11.89 ± 5.27 vs. 13.18 ± 3.24 and 13.10 ± 4.66; myocardial SNR: 8.20 ± 2.94 vs. 10.00 ± 3.86 and 8.64 ± 3.61; CNR: 7.35 ± 2.19 vs. 8.53 ± 1.94 and 6.79 ± 2.12; all *p* > 0.05). In contrast, SW‐tyGA‐UTE displayed significantly lower values for all three image quality metrics compared with all three gated protocols (all *p* < 0.01), with a blood pool SNR of 5.22 ± 1.18, myocardial SNR of 2.17 ± 0.45, and CNR of 2.87 ± 0.82. This pronounced degradation in the sliding‐window reconstruction reflects the inherent spatiotemporal trade‐off of undersampled dynamic imaging, and the within‐ROI metric further captures structured reconstruction artifacts such as streaking that arise from sparse radial sampling.

**FIGURE 5:**
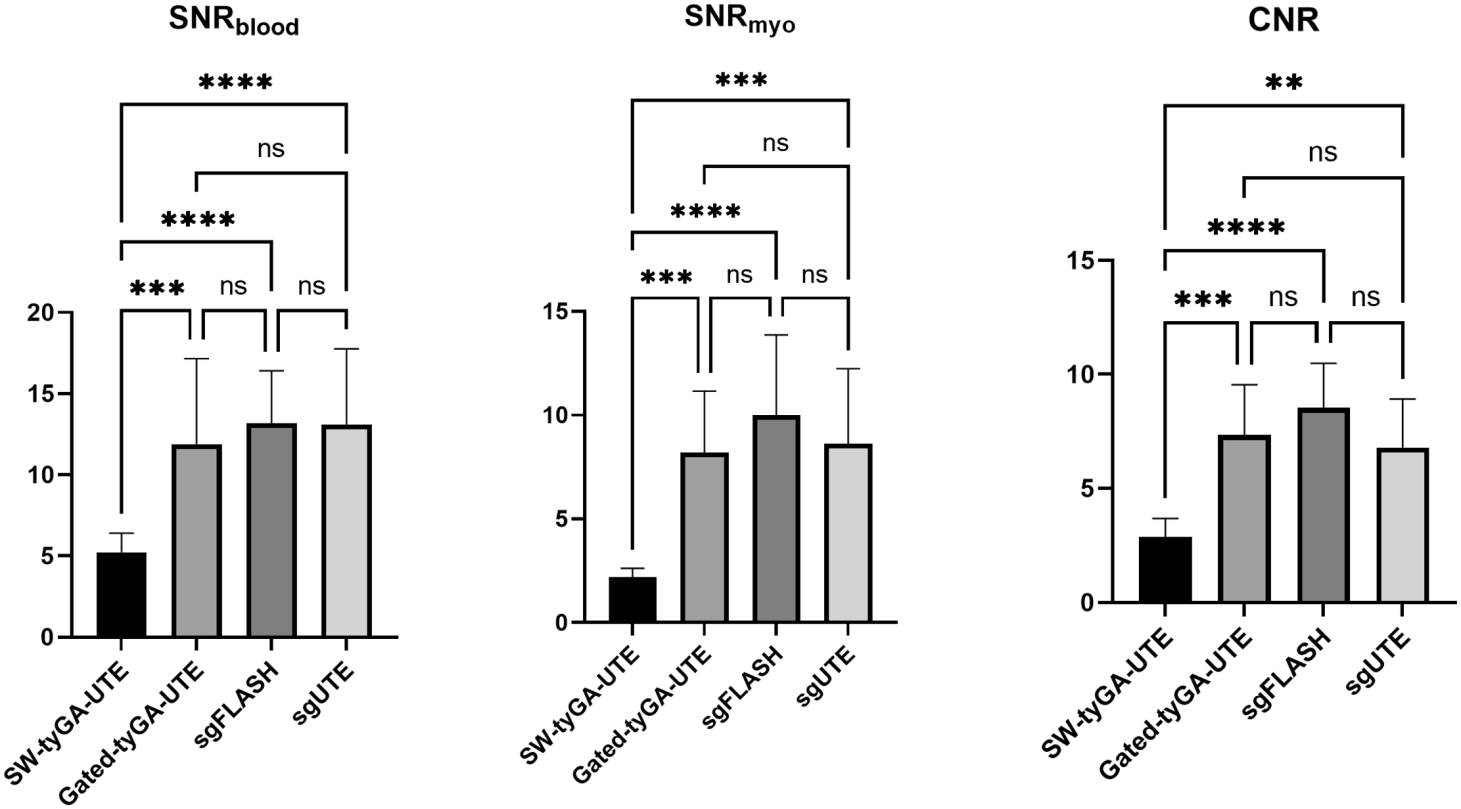
Quantitative comparison of left ventricular blood pool SNR, myocardial SNR, and myocardium‐blood CNR across all four imaging protocols, quantified using the image‐based within‐ROI noise estimation method. Gated tyGA‐UTE demonstrates nonsignificant differences in all three image quality metrics relative to sgFLASH and sgUTE (all *p* > 0.05). SW‐tyGA‐UTE yields significantly lower blood pool SNR, myocardial SNR, and CNR than the three gated sequences. ns = nonsignificant; **p < 0.01; ***p < 0.001; ****p < 0.0001.

These results confirm that Gated‐tyGA‐UTE delivers diagnostic‐quality image performance comparable to standard navigator‐gated sequences, whereas SW‐tyGA‐UTE trades off image quality to achieve ultra‐high temporal resolution for dynamic cardiac visualization.

## 4 Discussion

This study demonstrates the use of tyGA UTE sampling for high‐temporal resolution sliding‐window imaging as well as high‐fidelity self‐gated reconstruction. In direct comparison to the current standard self‐gated FLASH/UTE techniques, there were no statistically significant differences in the cardiac functional parameters observed with either approach, even though image quality was clearly impaired with the sliding‐window approach reconstructed with high temporal resolution of 18.8 ms.

Radial sampling, k‐space‐based self‐gating, and golden angle trajectories are well‐established individual techniques for small‐animal cardiac MRI, but they are rarely integrated into a single unified pipeline optimized for UHF. The tyGA trajectory minimizes gradient‐induced eddy currents [29] through smaller irrational angular increments that preserve flexible retrospective binning. Furthermore, UTE data readout reduces intraventricular flow‐related signal loss, as often observed in conventional echo‐base acquisitions most prominently during ventricular filling. This mitigation of flow‐related signal voids improves delineation of endocardial boundaries and may support more reliable quantitative assessment of valvular function, a meaningful advantage for studies focusing on ventricular remodeling or valve pathophysiology.

Compared with reported multi‐gating radial cardiac MRI methods [36], our protocol offers two specific strengths for 11.7‐T systems: inherent eddy‐current reduction via the tyGA trajectory and an all‐in‐one acquisition design that eliminates the need for dedicated navigator pulses or repeated sequential scans. This streamlined workflow reduces total anesthesia time and minimizes physiological variability between scans, which is particularly valuable for longitudinal studies and high‐throughput screening.

Motion correction and sampling efficiency remain central challenges for preclinical cardiac MRI given the rapid heart and respiratory rates of mice. In recent years, a range of advanced radial trajectories and motion correction strategies have been developed to further optimize small‐animal cardiac imaging. Novel radial variants, including swing golden angle (swingGA) trajectories and simulation‐optimized sampling patterns, have been proposed to better balance eddy current suppression and sampling uniformity [37]. For motion gating, beyond the conventional navigator‐based self‐gated approach used as the reference standard in this work, multiple advanced solutions have emerged, such as spatially resolved PolyGate for high‐throughput multi‐animal scanning [38], prospective ECG/respiratory gating, and fully self‐navigated radial reconstruction, each with distinct trade‐offs in field strength compatibility, acquisition efficiency, and implementation complexity. In parallel, simultaneous multi‐slice (SMS) techniques [39] combined with radial encoding have been demonstrated to boost scan throughput, whereas deep learning (DL) and machine learning (ML) [40] approaches have shown great promise for accelerated image reconstruction, denoising, and artifact mitigation in cardiac MRI.

Nevertheless, most of these emerging methods are optimized for specific experimental scenarios (e.g., multi‐animal population screening and lower magnetic field strengths) or require substantially increased computational and setup overhead. To date, few have been systematically validated for routine single‐subject quantitative cardiac imaging at 11.7‐T UHF. Navigator‐based self‐gated FLASH and UTE remain the most widely adopted benchmarks for robust, reproducible functional assessment in preclinical cardiac research, which justifies their use as reference standards in the present study. The proposed tyGA‐UTE enables single‐acquisition dual‐output reconstruction, and reduced anesthesia exposure (Data S1), addressing a practical need for longitudinal studies and detailed phenotypic characterization. The workflow extracts cardiac and respiratory motion signals directly from the central k‐space (k_0_) of continuously acquired tyGA data, requiring no external sensors or dedicated navigator modules. The uniform k‐space filling, independent on the number of projections with the tyGA trajectory, ensures flexible choice of number or projections throughout the acquisition, which is an excellent prerequisite for self‐gated reconstructions. The equivalent functional accuracy and image quality between gated tyGA‐UTE and navigator‐gated references at substantial shorter acquisition time confirm that this data‐driven k_0_ self‐gating approach strikes a favorable balance of robustness, scan efficiency, and computational simplicity for routine UHF cardiac imaging.

All left ventricular functional parameters derived from the tyGA‐UTE workflow demonstrated excellent intra‐ and inter‐observer reproducibility, with ICC values above 0.90 across all metrics. Notably, ejection fraction exhibited the highest repeatability in both analyses. This high stability of EF is clinically relevant, as it is the most widely used endpoint for evaluating systolic function in preclinical studies.

In contrast, SV showed the widest limits of agreement and the largest coefficient of variation among all parameters. This pattern can be explained by the physiology of the murine cardiac cycle: The end‐systolic phase is very short in mice, and minor differences in the timing of the selected systolic frame or small deviations in endocardial contouring can introduce proportionally larger variations in SV measurements. Despite this slightly higher variability, all reproducibility metrics remain within acceptable ranges for preclinical cardiac MRI, supporting the reliability of quantitative comparisons across reconstruction modes and sequences.

The substantial gap in SNR and CNR between dynamic and gated tyGA reconstructions arises from inherent temporal–spatial trade‐offs of undersampled sliding‐window reconstruction. To achieve ultra‐fast frame rates (~18.88 ms), the dynamic reconstruction pipeline groups far fewer radial spokes into each temporal bin, leading to severe undersampling and elevated background levels. In comparison, the Gated‐tyGA‐UTE scheme considers all projections across complete cardiac and respiratory cycles, delivering sufficient k‐space sampling density to match the image quality of standard sgFLASH and sgUTE.

To address the known limitation of conventional noise quantification for CS‐reconstructed MRI, we adopted a within‐ROI noise estimation approach that quantifies signal variation directly within tissue regions of interest. This approach incorporates structured reconstruction artifacts (e.g., streaking from radial undersampling in SW‐tyGA‐UTE) into the noise metric, providing a clinically relevant measure of effective image quality for visual assessment and functional delineation, and strengthens the conclusion that Gated‐tyGA‐UTE delivers diagnostic‐quality cine images comparable to established standard protocols, despite being derived from a single continuous acquisition without dedicated navigator pulses.

Although the sliding‐window tyGA pipeline sacrifices image quality and presents more pronounced reconstruction‐related noise, its ultra‐high temporal resolution remains uniquely valuable for visualizing rapid beat‐to‐beat cardiac motion in murine models (Data S5), a critical strength that cannot be reproduced by slower conventional gated acquisitions. The dual outputs from one unified tyGA scan therefore deliver complementary strengths: high‐fidelity quantitative analysis via gated reconstruction and dynamic functional visualization via the sliding‐window pipeline, each with predictable, well‐understood image quality trade‐offs.

In addition, inherent TR differences across sequences (caused by navigator acquisition in conventional self‐gated FLASH/UTE) lead to different steady‐state magnetization levels, which is an unavoidable confounding factor for direct quantitative SNR comparison. For this reason, we emphasize that SNR/CNR results are used for intra‐sequence performance evaluation rather than absolute cross‐sequence quantification. Additionally, coil sensitivity maps in this work were self‐calibrated from central radial k‐space data. This standard radial self‐calibration method produces stable and accurate sensitivity profiles, which have negligible impact on reconstruction performance and quantitative SNR/CNR measurements.

Several limitations warrant consideration. First, the high‐temporal‐resolution sliding‐window mode exhibits significantly reduced SNR and CNR, restricting its use to qualitative dynamic visualization rather than precise quantitative analysis‐an inherent trade‐off of ultrafast radial accelerated imaging. Second, compressed sensing reconstruction for sliding‐window data requires substantially longer computation time than vendor‐supplied navigator‐based reconstructions, though ongoing advances in computing hardware and algorithms are progressively addressing this practical barrier. Third, all experiments were conducted in healthy wild‐type mice; the robustness of self‐gating signal extraction and artifact performance in pathological models (e.g., arrhythmia, myocardial infarction, and hypertrophy) remains to be validated.

Further optimization can be pursued in multiple directions. Parallel imaging techniques or advanced low‐rank/sparse reconstruction models could be integrated to mitigate the SNR limitation of the sliding‐window mode. Combining the tyGA pipeline with simultaneous multi‐slice imaging or DL‐accelerated reconstruction would further improve scan and post‐processing efficiency. Most importantly, validation across a broader range of cardiovascular disease models is needed to establish the generalizability of the workflow. Beyond ventricular function, the “one acquisition, multiple outputs” concept could be extended to late gadolinium enhancement and parametric mapping (T1, T2) with appropriate sequence modifications, substantially increasing information yield per scan session.

In summary, the tyGA‐UTE integrated workflow provides a flexible, efficient, and robust solution for UHF small‐animal cardiac MRI. By unifying high‐quality cine quantification and high‐frame‐rate sliding‐window imaging within a single acquisition, it reduces experimental complexity and physiological variability, offering practical benefits for preclinical cardiovascular research.

## 5 Conclusions

The tiny golden angle radial trajectory provides high flexibility and efficiency for multimodal retrospective reconstruction. Based on this technique, we validated an integrated workflow for murine cardiac MRI at 11.7 T. A single continuous tyGA acquisition can be flexibly reconstructed into both high‐temporal‐resolution sliding‐window retrospective dynamic images and high‐quality cardiorespiratory self‐gated cine images. This integrated scheme combines separate imaging protocols into one efficient procedure, avoiding repeated scans, reducing animal physiological variability and improving the throughput of preclinical cardiac experiments. This practical workflow optimization has good application prospects for longitudinal research and drug screening. In future work, this “one acquisition, multiple outputs” strategy can be further extended to cardiac parametric mapping and translational studies in large animal models.

## Author Contributions

**Hao Li:** writing – original draft, writing – review and editing, conceptualization, methodology, investigation, data curation, software, formal analysis, validation, visualization. **Patrick Metze:** writing – review and editing, conceptualization, methodology, software, data curation, formal analysis. **Pengxi Han:** writing – review and editing, investigation, data curation, validation. **Benzheng Wei:** writing – review and editing, conceptualization, supervision, funding acquisition. **Volker Rasche:** writing – review and editing, conceptualization, supervision, resources, project administration.

## Funding

This work was supported by the Youth Fund Cultivation Project of Shandong First Medical University (Grant No. 202201‐081), the Youth Program of Shandong Provincial Natural Science Foundation (Grant No. ZR2023QH027), and the Cultivation Fund of the First Affiliated Hospital of Shandong First Medical University (Grant No. QYPY2022NSFC0807).

## Conflicts of Interest

The authors declare no conflicts of interest.

## Supporting information


**Data S1:** Full workflow time consumption comparison across three imaging protocols.
**Data S2:** Standardized multi‐plane imaging planning workflow for murine cardiac MRI. All timing efficiency comparisons between separate multi‐scan FLASH/UTE and unified single tyGA acquisition were performed using this identical standardized slice layout, including 1 × 2CH, 1 × 4CH, and 8 contiguous full‐ventricle SAX slices (10 total slices).
**Data S3:** Bland–Altman plots displaying 95% limits of agreement for intra‐observer reproducibility (two repeated measurements S1 and S2) of left ventricular functional metrics acquired with the tyGA‐UTE workflow, including high‐temporal‐resolution sliding‐window retrospective dynamic reconstruction and self‐gated cine reconstruction. Vertical axes show relative measurement variability, calculated as the absolute difference between paired observations normalized to their respective mean value. CI, confidence interval; EDV, end‐diastolic volume; ESV, end‐systolic volume; ICC, intraclass correlation coefficient; LVMED, end‐diastolic left ventricular mass; LVMES, end‐systolic left ventricular mass; SV, stroke volume; EF, ejection fraction.
**Data S4:** Bland–Altman plots displaying 95% limits of agreement for inter‐observer reproducibility (measurements S1 and S2) of left ventricular functional metrics acquired with the tyGA‐UTE workflow, including high‐temporal‐resolution sliding‐window retrospective dynamic reconstruction and self‐gated cine reconstruction. Vertical axes show relative measurement variability, calculated as the absolute difference between paired observations normalized to their respective mean value. CI, confidence interval; EDV, end‐diastolic volume; EF, ejection fraction; ESV, end‐systolic volume; ICC, intraclass correlation coefficient; LVMED, end‐diastolic left ventricular mass; LVMES, end‐systolic left ventricular mass; SV, stroke volume.
**Data S5:** Continuous tyGA acquisition with simultaneous administration of contrast agent. (a) The signal intensities over time with medium temporal resolution. (b) The in (a) indicated range during actual CA injection with improved temporal resolution. Please note the drop in signal intensity in the right heart due to high CA concentration in the blood directly after injection. (c) Gated reconstruction from the same datasets, reconstructed from data acquired prior to injection, right after injection and during the wash‐out phase. Please note that the pre/post injection phase can be clearly identified by the increase of the gating signal.

**Data S6:** Supporting Information.

## Data Availability

The datasets generated and analyzed are available from the corresponding author upon reasonable request.
