# Supplementary material for: Radial Tiny Golden Angle UTE (tyGA‐UTE) for Cardiac MRI in the Small Animal Model
Source: NMR Biomed. 2026 Oct 14;39(11):e70418. doi: 10.1002/nbm.70418 (PMC13669412; doi:10.1002/nbm.70418)
Supplement: Supplementary file 1 [file NBM-39-e70418-s001.docx]

Supplementary Material S1. Full workflow time consumption comparison across three imaging protocols

|  | **Self-gated FLASH** | **Self-gated UTE** | **tyGA-UTE** |
| --- | --- | --- | --- |
| Mandatory pre-scan localization | 49 s | 49 s | 49 s |
| Total in-bore sequence scan time | 26 min 13s | 34min 18 s | 12 min 45s |
| Offline reconstruction | <30 s (auto vendor recon) | <30 s (auto vendor recon) | $\approx$10min |
| Total workflow time | 27 min 32s | 35min 37s | 23 min 34s |
| Continuous animal anesthesia duration | 27 min 12s | 35min 17s | 13min 34s |

Note: All reconstruction for tyGA runs offline after the animal is removed from the scanner and anesthesia is terminated. The high-temporal-resolution sliding-window reconstruction uses binsize=8, stepsize=4, generating 200 dynamic frames per slice; self-gated cine reconstruction outputs phase-binned cardiac motion-resolved images.

Supplementary Material S2. Standardized multi-plane imaging planning workflow for murine cardiac MRI.All timing efficiency comparisons between separate multi-scan FLASH/UTE and unified single tyGA acquisition were performed using this identical standardized slice layout, including 1×2CH, 1×4CH, and 8 contiguous full-ventricle SAX slices (10 total slices).

Supplementary Material S3: Bland-Altman plots displaying 95% limits of agreement for intra-observer reproducibility (two repeated measurements S1 and S2) of left ventricular functional metrics acquired with the tyGA-UTE workflow, including high-temporal-resolution sliding-window retrospective dynamic reconstruction and self-gated cine reconstruction. Vertical axes show relative measurement variability, calculated as the absolute difference between paired observations normalized to their respective mean value. Abbreviations: EDV, end-diastolic volume; ESV, end-systolic volume; SV, stroke volume; EF, ejection fraction; LVMED, end-diastolic left ventricular mass; LVMES, end-systolic left ventricular mass; ICC, intraclass correlation coefficient; CI, confidence interval.

Supplementary Material S4: Bland-Altman plots displaying 95% limits of agreement for inter-observer reproducibility (measurements S1 and S2) of left ventricular functional metrics acquired with the tyGA-UTE workflow, including high-temporal-resolution sliding-window retrospective dynamic reconstruction and self-gated cine reconstruction. Vertical axes show relative measurement variability, calculated as the absolute difference between paired observations normalized to their respective mean value. Abbreviations: EDV, end-diastolic volume; ESV, end-systolic volume; SV, stroke volume; EF, ejection fraction; LVMED, end-diastolic left ventricular mass; LVMES, end-systolic left ventricular mass; ICC, intraclass correlation coefficient; CI, confidence interval.

Supplementary Material S5. Continuous tyGA acquisition with simultaneous administration of contrast agent. a) shows the signal intensities over time with medium temporal resolution; b) shows the in a) indicated range during actual CA injection with improved temporal resolution. Please note the drop in signal intensity in the right heart due to high CA concentration in the blood directly after injection; c) shows gated reconstruction from the same data sets, reconstructed from data acquired prior to injection, right after injection and during the wash-out phase. Please note that the pre/post injection phase can be clearly identified by the increase of the gating signal.
